# Potency of Olorofim (F901318) Compared to Contemporary Antifungal Agents against Clinical Aspergillus fumigatus Isolates and Review of Azole Resistance Phenotype and Genotype Epidemiology in China

**DOI:** 10.1128/AAC.02546-20

**Published:** 2021-04-19

**Authors:** Huilin Su, Min Zhu, Clement Kin-Ming Tsui, Henrich van der Lee, Marlou Tehupeiory-Kooreman, Jan Zoll, Tobias Engel, Li Li, Junhao Zhu, Zihan Lu, Qiangqiang Zhang, Paul E. Verweij, Shuwen Deng

**Affiliations:** a Department of Dermatology, Huashan Hospital, Fudan University, Shanghai, China; b Department of Medical Microbiology and Center of Expertise in Mycology Radboudumc/CWZ, Radboud University Medical Center, Nijmegen, The Netherlands; c Department of Dermatology, The First Affiliated Hospital of Sun Yat-sen University, Guangzhou, China; d Department of Pathology, Sidra Medicine, Doha, Qatar; e Department of Pathology and Laboratory Medicine, Weill Cornell Medicine-Qatar, Doha, Qatar; f Division of Infectious Diseases, Faculty of Medicine, University of British Columbia, Vancouver, British Columbia, Canada; g Center for Infectious Diseases Research, Diagnostics and Laboratory Surveillance, National Institute for Public Health and the Environment (RIVM), Bilthoven, the Netherlands; h Department of Medical Microbiology, The People's Hospital of Suzhou New District, Suzhou, Jiangsu, China

**Keywords:** olorofim, *Aspergillus fumigatus*, antifungal susceptibility, azole resistance, genetic diversity, China

## Abstract

Triazole resistance in Aspergillus fumigatus is an increasing worldwide problem that causes major challenges in the management of aspergillosis. New antifungal drugs are needed, with novel targets, that are effective in triazole-resistant infection. In this study, we retrospectively evaluated the potency of the novel drug olorofim compared to contemporary antifungal agents against 111 clinical A. fumigatus isolates collected from Huashan Hospital, Shanghai, China, using EUCAST methodology, and we reviewed the literature on triazole-resistant A. fumigatus (TRAF) published between 1966 and 2020 in China. Olorofim was active *in vitro* against all tested A. fumigatus isolates, with a MIC_90_ of 0.031 mg/liter (range, 0.008 to 0.062 mg/liter). For 4 triazole-resistant A. fumigatus isolates, the olorofim MIC ranged between 0.016 and 0.062 mg/liter. The reported rates of TRAF in China are 2.5 to 5.56% for clinical isolates and 0 to 1.4% for environmental isolates. TR_34_/L98H/S297T/F495I is the predominant resistance mechanism, followed by TR_34_/L98H. Non-TR-mediated TRAF isolates, mostly harboring a *cyp51A* single point mutation, showed greater genetic diversity than TR-mediated resistant isolates. Resistance due to TR_34_/L98H and TR_34_/L98H/S297T/F495I mutations among TRAF isolates might have evolved from separate local isolates in China. Continuous isolation of TRAF in China underscores the need for systematic resistance surveillance as well as the need for novel drug targets, such as olorofim.

## Abstract (precis)

Triazole resistance in Aspergillus fumigatus is an increasing worldwide problem that causes major challenges in the management of aspergillosis. New antifungal drugs are needed, with novel targets, that are effective in triazole-resistant infection.

## INTRODUCTION

Invasive aspergillosis (IA) in immunocompromised patients results in substantial morbidity and mortality (1). More than 40 *Aspergillus* species have been reported as causative agents of IA. Aspergillus fumigatus is the most common etiological agent of invasive and chronic pulmonary aspergillosis (1). Two classes of antifungal agents are licensed for the primary therapy of IA, namely, the triazoles and the polyene amphotericin B. Currently, triazole antifungals are recommended as the first choice for prophylaxis and treatment of aspergillosis (1). However, since the first report of triazole resistance in 1997 (2), many centers/hospitals around the world have reported resistance. Furthermore, voriconazole-resistant IA was found to be associated with treatment failure and excess mortality, which threatens the current treatment strategy for this pathogen (3, 4).

The most common mechanism of triazole resistance is associated with mutations in the *cyp51A* gene, which encodes the protein targeted by the triazoles (5). Apparently, the mutant allele has spread throughout the A. fumigatus population and, thus, has been reported worldwide from patients as well as from the environment. In addition, several point mutations, such as G54, G138, and M220, intervene with the docking of azole drugs to CYP51A protein and render an azole-resistant phenotype (3). Rates of azole resistance in A. fumigatus vary extensively among countries and centers worldwide (6–9), and in many countries the presence and frequency of azole resistance remain unknown. Multiple factors contribute to the observed variation in resistance frequency, including sample size, method of resistance detection, and geographical differences (10). The overall azole resistance rates ranged from 0 to 27.8% in different surveys (11–13). Since the spread of antifungal drug resistance has shown no signs of diminishing and new resistance mechanisms continue to emerge (14), understanding the genetic variability and relationship among resistant isolates from various parts of the world is of major importance. Azole resistance surveillance programs are scarce, and in China data on the prevalence of azole-resistant A. fumigatus are very limited. A few Chinese reports on triazole resistance in A. fumigatus are available, although most reports are from restricted geographic areas and include only a modest number of isolates (7, 13, 15–19). Furthermore, the genetic relationship and variability of azole-resistant isolates of A. fumigatus in China remain unclear.

The clinical development of new antifungal drug classes is critical to overcoming current and future challenges in the management of *Aspergillus* diseases. Olorofim (formerly F901318), a leading representative of a novel class of drug belonging to orotomides, is an antifungal drug in clinical development that demonstrates excellent potency against a broad range of dimorphic and filamentous fungi, and it targets an important enzyme for pyrimidine biosynthesis, dihydroorotate dehydrogenase (20). The drug has *in vitro* activity against *Aspergillus* species and other difficult-to-treat molds, including *Scedosporium* and *Lomentospora* species, but lacks activity against *Candida*, *Cryptococcus*, and *Mucorales* species due to differences in drug target affinity (20–22). For *Aspergillus* species specifically, Buil et al. demonstrated *in vitro* activity against azole wild-type (WT) isolates as well as azole-resistant *cyp51A* mutant A. fumigatus isolates, also including a limited number of other *Aspergillus* species originating from the Netherlands (20).

We aimed to evaluate the potency of olorofim against a large set of clinical A. fumigatus isolates collected from China and compare the activity with that of contemporary antifungal agents. We further reviewed the prevalence of azole resistance and underlying *cyp51A* mutations in clinical and environmental A. fumigatus isolates in China.

## RESULTS

The *in vitro* activities of olorofim and comparator agents against 111 clinical A. fumigatus isolates from China are shown in Table 1. The ofolorofim MICs ranged between 0.008 and 0.062 mg/liter, which were, in general, lower than the MICs of the azoles and amphotericin B. Compared with echinocandins, olorofim showed MICs (MIC_90_, 0.031 mg/liter; modal MIC, 0.031 mg/liter; *n* = 70) similar to those of anidulafungin (90% minimum effective concentration [MEC_90_], 0.031 mg/liter; modal MEC, 0.016 mg/liter; *n* = 67), slightly higher than those of micafungin (MEC_90_, 0.016 mg/liter; modal MEC, 0.008 mg/liter; *n* = 61), and significantly lower than those of caspofungin (MEC_90_, 0.5 mg/liter; modal MEC, 0.25 mg/liter; *n* = 67). Posaconazole (modal MICs, 0.062 mg/liter; *n* = 70) exhibited the lowest modal MICs of the azoles in this study, followed by itraconazole (0.25 mg/liter; *n* = 53), voriconazole (0.5 mg/liter; *n* = 85), and isavuconazole (0.5 mg/liter; *n* = 83). Amphotericin B had relatively higher modal MICs (0.5 mg/liter; *n* = 97).

**TABLE 1:** MIC/MEC ranges and geometric means, modal MIC/MEC, and distribution of MIC/MEC of 111 clinical A. fumigatus isolates from China for 9 antifungal agents^*a*^

Antifungal agent	MIC/MEC range (mg/liter)	Geometric mean (mg/liter)	MIC/MEC_50_ (mg/liter)	MIC/MEC_90_ (mg/liter)	No. of isolates with MIC/MEC of:														No. (%) of resistant strains
					0.002	0.004	0.008	0.016	0.031	0.062	0.125	0.25	0.5	1	2	4	8	16	
Olorofim	0.008–0.062	0.025	0.031	0.031	3	34	70	4
Itraconazole	0.125–16	0.373	0.25	0.5	7	53	47	4	4 (3.60)
Voriconazole	0.25–8	0.500	0.5	1	14	85	10	2*	0
Posaconazole	0.031–1	0.078	0.062	0.125	7	70	29	3*	2	2 (1.80)
Isavuconazole	0.25–16	0.574	0.5	1	6	83	19	2*	1	3 (2.70)
Amphotericin B	0.125–1	0.529	0.5	1	1	1	97	12
Anidulafungin	0.016–0.062	0.021	0.016	0.031	67	43	1
Caspofungin	0.062–0.5	0.271	0.25	0.5	3	11	67	30
Micafungin	0.002–0.062	0.009	0.008	0.016	1	14	61	30	2	3

a For modal MIC/MEC, values in boldface indicate the most frequent MIC/MEC, underlined values indicate the resistant isolates, and values with an asterisk indicate the strains in the area of technical uncertainty (ATU). MICs are shown for amphotericin B, itraconazole, voriconazole, posaconazole, isavuconazole, and olorofim; MECs are shown for anidulafungin, caspofungin, and micafungin.

*In vitro* activities of olorofim and comparator agents against 4 TRAF isolates are shown in Table 2. Four TRAF isolates were highly resistant to itraconazole (MIC, >16 mg/liter), and two isolates were in the area of technical uncertainty (ATU) of voriconazole (MIC, 2 mg/liter), posaconazole (MIC, 0.25 mg/liter), and isavuconazole (MIC, 2 mg/liter). Isolate 247-34 was resistant to both posaconazole (MIC, 1 mg/liter) and isavuconazole (MIC > 16 mg/liter), and isolate 247-32 was resistant to posaconazole (MIC, 1 mg/liter). A resistance mutation was detected in *cyp51A* of two isolates, G54V in isolate 247-32 and TR_34_/L98H/S297T/F495I in isolate 247-34 (Table 2).

**TABLE 2:** MIC/MEC values and *cyp51A* gene mutation type of four azole-resistant A. fumigatus isolates detected in this study^*a*^

Isolate no.	MIC/MEC (mg/liter)									Mutation in *cyp51A*
	Olo	Itra	Vori	Posa	Isa	AmB	Anid	Cas	Mica	
247-11	0.062	>16	2*	0.25*	2*	0.5	0.031	0.25	0.008	WT
247-20	0.031	>16	2*	0.25*	2*	0.5	0.016	0.25	0.008	WT
247-32	0.016	>16	0.5	1	0.5	0.5	0.016	0.25	0.008	G54V
247-34	0.031	>16	0.5	1	>16	0.5	0.016	0.5	0.008	TR_34_/L98H/S297T/F495I

a Abbreviations: amphotericin B, AmB; itraconazole, Itra; voriconazole, Vori; posaconazole, Posa; isavuconazole, Isa; olorofim, Olo; anidulafungin, Anid; caspofungin, Cas; micafungin, Mica. MICs are shown for amphotericin B, itraconazole, voriconazole, posaconazole, isavuconazole, olorofim; MECs are shown for anidulafungin, caspofungin, micafungin. Asterisks indicate values in the area of technical uncertainty.

Among the four TRAF isolates detected in this study, MIC values of olorofim (range, 0.016 to 0.062 mg/liter) were in the same range as those observed for the azole WT isolates. The lowest olorofim MIC was seen in isolate 247-32 with G54V, and the highest MIC was 0.062 mg/liter, for azole-resistant A. fumigatus isolates with the WT *cyp51A* gene.

### Microsatellite typing.

**FIG 1:**
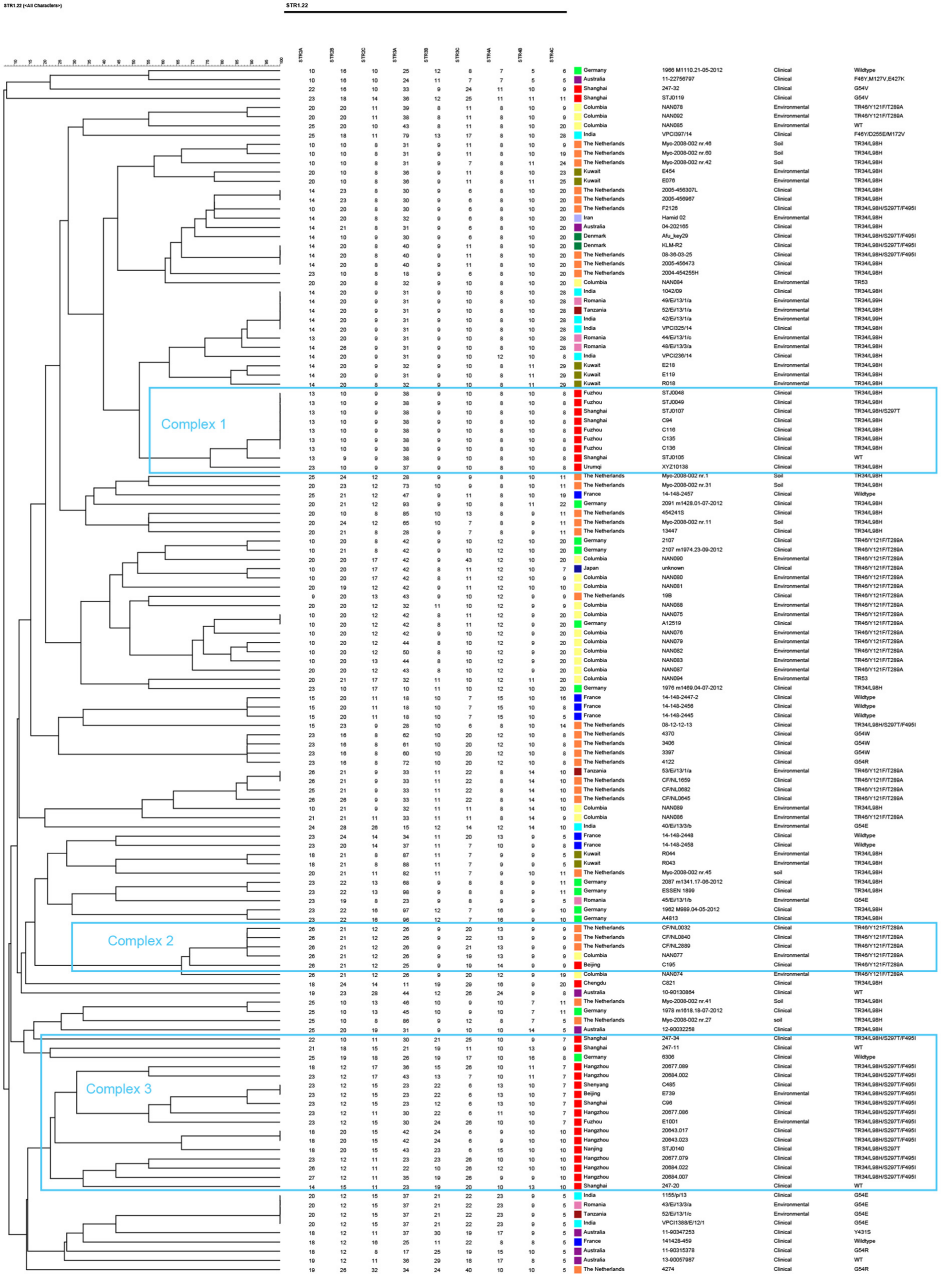
Genotypic relationship of Chinese azole-resistant A. fumigatus isolates (clinical, *n* = 27; environmental, *n* = 2) with isolates from Columbia (environmental, *n* = 19), Denmark (clinical, *n* = 2), France (clinical, *n* = 7), Germany (clinical, *n* = 12), India (clinical, *n* = 6; environmental, *n* = 2), Iran (clinical, *n* = 1), Japan (clinical, *n* = 1), Kuwait (environmental, *n* = 7), the Netherlands (clinical, *n* = 21; environmental, *n* = 9), Romania (environmental, *n* = 5), Tanzania (environmental, *n* = 3), and Australia (clinical, *n* = 7).

The genetic polymorphism of TRAF isolates from China and outside China was studied using short tandem repeat (STR) typing (Fig. 1). Multiple distinct clusters can be identified based on microsatellite markers. STR typing of 29 Chinese TRAF isolates revealed 21 distinct genotypes distributed among environmental and clinical isolates that represented a major complex of the TRAF isolates disseminating all around the world.

Three microsatellite complexes (MCs) among the 21 *cyp51A* mutant genotypes of Chinese TRAF were recognizable, representing three distinct complexes of TRAF (Fig. 1). Seven isolates with TR_34_/L98H in complex 1 were clonal and shared all nine loci except for two isolates, with one difference in one repeat at a single locus (2B) and the other at three loci (2A, 2B, and 3A). Thirteen isolates with mutation TR_34_/L98H/S297T/F495I in complex 3 were highly polymorphic and different from the isolates with the same mutation from the Netherlands and Denmark, which clustered in complex 1. Among 13 polymorphic genotypes observed in TR_34_/L98H/S297T/F495I isolates, an identical allelic profile was observed in a clinical (isolate C485) and an environmental isolate (isolate E739). One isolate with TR_46_/Y121F/T289A from Beijing was clustered in a complex group with isolates harboring TR_46_/Y121F/T289A from the Netherlands and Columbia.

**FIG 2:**
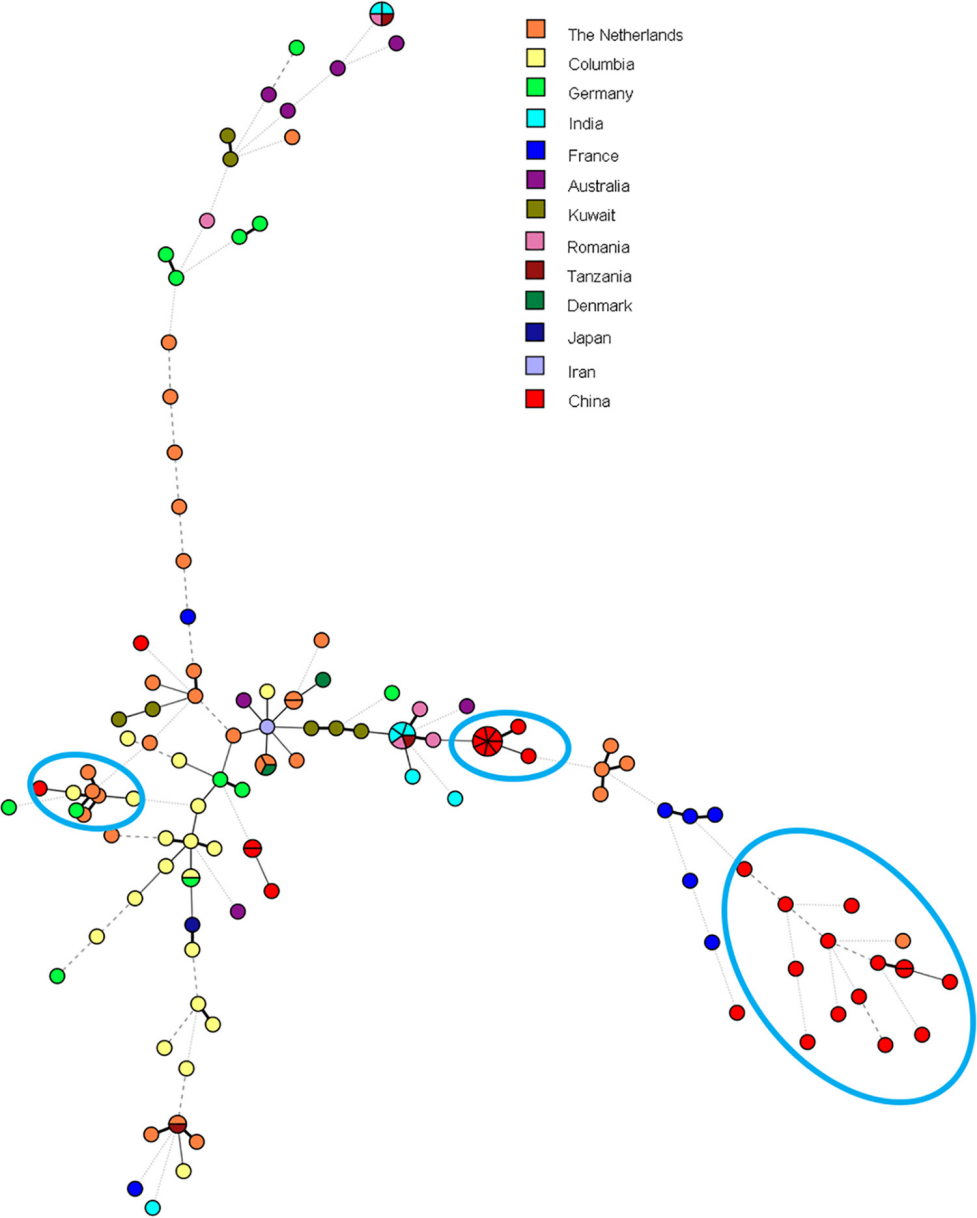
Minimum spanning tree of 131 azole-resistant A. fumigatus isolates based on all nine microsatellite markers of STR typing.

The genotypic relationships among Chinese and global isolates were also inferred from the minimum spanning tree (Fig. 2). High genetic variability was observed among A. fumigatus isolates, which was not associated with the country and continent of origin. TRAF in China showed divergence in genetic variability as well.

### Literature review.

Our literature review resulted in 8 publications reporting azole resistance in A. fumigatus in China (7, 13, 15–18, 23, 24). TRAF was first reported in 2004 involving two isolates with single resistance point mutations (Table 3). Resistance involving TRs was first reported in 2011 and has since been the dominant resistance variant in China. Azole resistance rates in A. fumigatus isolates ranged from 2.5% to 5.56% for clinical isolates and 0 to 1.4% for environmental isolates, and the rate of azole resistance in A. fumigatus isolates in the current study was 3.6% (four of 111 isolates), which was within this range. Origin, source, and antifungal susceptibility profiles of TR-mediated azole-resistant A. fumigatus isolates in China from 2004 to 2019 are summarized in Table 4.

**TABLE 3:** Overview of TRAF isolates harboring mutations in the *cyp51A* gene from clinical and environmental sources in China, 2004 to 2019

Yr published	Resistance mechanism	Source	Resistance rate [no. of resistant isolates/no. of isolates tested (%)]	Antifungal susceptibility testing methods	Reference or source
2020	G54V (*n* = 1); TR_34_/L98H/S297T/F495 (*n* = 1); WT (*n* = 2)	Clinical	4/111 (3.60)	EUCAST 9.3.1	Current study
2017	M220I (*n* = 1); TR_34_/L98H (*n* = 2); WT (*n* = 1)	Clinical	4/126 (3.17)	EUCAST 9.1	Zhang et al. (23)
2017	TR_34_/L98H (*n* = 3); TR_34_/L98H/S297T (*n* = 2); G54V (*n* = 1); WT (*n* = 1)	Clinical	7/159 (4.40)	CLSI M38-A2	Deng et al. (7)
2017	TR_46_/Y121F/T289A (*n* = 2); TR_34_/L98H/S297T/F495I (*n* = 1)	Environmental	3/144 (2.08)	CLSI M38-A2	Ren et al. (24)
2016	TR_34_/L98H (*n* = 5); TR_34_/L98H/S297T/F495I (*n* = 2); TR_46_/Y121F/T289A (*n* = 1)	Clinical	8/317 (2.5)	EUCAST 9.3	Chen et al. (15)
TR_34_/L98H/S297T/F495I (*n* = 2)	Environmental	2/144 (1.4)
2015	TR_34_/L98H/S297T/F495I (*n* = 2); G432A (*n* = 1); TR_34_/L98H (*n* = 1)	Clinical	4/72 (5.56)	EUCAST 9.1	Liu et al. (16)
2014	Environmental	0/51 (0.00)	CLSI M38-A2	Wang et al. (18)
2011	TR_34_/L98H/S297T/F495I (*n* = 8); WT (*n* = 2)	Clinical	24 (above ECV^*a*^); 10 (ITR or VORI, >2 μg/ml)	CLSI M38-A2	Lockhart et al. (13)
2004	M220I (*n* = 1); G54R (*n* = 3)	Clinical	4/6^*b*^	NCCLS M38-A	Chen et al. (17)

a Epidemiological cutoff values (ECV) are 1 μg/ml for itraconazole and voriconazole and 0.25 μg/ml for posaconazole. The total number of strains tested is not reported.

b Six clinical strains isolated from the same patient.

**TABLE 4:** Origin, source, and antifungal susceptibility of TR-mediated azole-resistant A. fumigatus isolates in China originating from the literature, 2004 to 2020^*a*^

Resistance mechanism	Strain ID no.	Region	Source	MIC, mg/liter		
				Itra	Vori	Posa
TR_34_/L98H/S297T/F495I (*n* = 16)	247-34	Shanghai	Clinical	>16	0.5	1
51	Zhejiang	Environmental	8–16	1	0.5
C96	Shanghai	Clinical	>16	1	0.5
C485	Shenyang	Clinical	>16	2	1
E739	Beijing	Environmental	>16	2	0.5
E1001	Fuzhou	Environmental	>16	1	0.5
SHJT42b	Fuzhou	Clinical	16	2	0.5
NJ21-76	Nanjing	Clinical	16	0.25	0.5
20643.017	Hangzhou	Clinical	16	2	2
20643.023	Hangzhou	Clinical	16	2	2
20677.079	Hangzhou	Clinical	16	1	1
20677.086	Hangzhou	Clinical	16	2	2
20677.089	Hangzhou	Clinical	16	4	2
20684.002	Hangzhou	Clinical	16	2	2
20684.007	Hangzhou	Clinical	16	2	2
20684.022	Hangzhou	Clinical	16	2	1
TR_34_/L98H *n* = 11	AF.44	Nanjing	Clinical	>8	4	0.5
AF.98	Nanjing	Clinical	>8	2	0.25
STJ0048	Fuzhou	Clinical	>16	1	1
STJ0049	Fuzhou	Clinical	>16	1	1
XJ138	Urumqi	Clinical	16	2	0.5
C94	Shanghai	Clinical	≥16	2	1
C116	Fuzhou	Clinical	≥16	4	0.5
C135	Fuzhou	Clinical	≥16	2	0.5
C136	Fuzhou	Clinical	≥16	2	0.5
C821	Chengdu	Clinical	≥16	4	1
SHJT40	Shanghai	Clinical	16	1	0.5
TR_34_/L98H/S297T *n* = 2	STJ0107	Shanghai	Clinical	>16	0.5	1
STJ0140	Nanjing	Clinical	>16	0.5	1
TR_46_/Y121F/T289A *n* = 3	15	Zhejiang	Environmental	0.5	8–16	0.25
44	Zhejiang	Environmental	0.5	8–16	0.25
C195	Beijing	Clinical	1	≥16	0.5

a These data originated from the literature (7, 13, 15–18, 23, 24). Abbreviations: Itra, itraconazole; Vori, voriconazole; Posa, posaconazole.

## DISCUSSION

In this study, we show that olorofim exhibits potent *in vitro* activity against 111 clinical A. fumigatus isolates, including TRAF from China. For the determination of wild-type upper limits (WT-UL) of visual values of A. fumigatus susceptibility to olorofim, we followed the 0.25 mg/liter value, as proposed by Jørgensen et al. (25). Olorofim MICs were low against 111 A. fumigatus isolates (modal MIC, 0.031 mg/liter; MIC range, 0.008 to 0.062 mg/liter), indicating that all MICs were within the range of the WT population. The observed MIC ranges are similar to those reported in previous reports from other geographic areas (19–21). The potency of olorofim was superior to that of triazoles and amphotericin B and comparable to those of three echinocandins tested. No substantial implications of the specific azole resistance mechanism for the activity of olorofim were demonstrated.

In an itraconazole-resistant A. fumigatus isolate with a G54V mutation, obtained from a patient undergoing high-dose itraconazole therapy, olorofim was 5- to 6-fold more potent than voriconazole and posaconazole, respectively. Furthermore, in an isolate harboring TR_34_/L98H/S297T/F495I, olorofim was 4-, 5-, and 9-fold more potent than voriconazole, posaconazole, and isavuconazole, respectively. Olorofim was also more active than voriconazole and isavuconazole against the two other TRAF isolates with WT *cyp51A* genes. These findings confirm previous reports (20, 22, 26) and indicate that triazole resistance does not affect olorofim activity, as olorofim MICs of these isolates are within the olorofim WT population (25).

The rate of azole resistance in A. fumigatus isolates in China (2.5% to 5.56%) is around the lowest border compared to the high prevalence in Europe, including the United Kingdom (6.6 to 27.8%), the Netherlands (3.1 to 4.6%), and Germany (3.2%) (27–30) The first report on the occurrence of TRAF isolates originated from China during 2008 to 2009 from the ARTEMIS global sentinel surveillance program, which demonstrated the TR_34_/L98H/S297T mechanism in 27.5% (8/29) of A. fumigatus isolates (13) (Table 3). Our study, reviewing Chinese TRAF isolates from 2004 to 2019, confirmed that TR_34_/L98H/S297T/F495I (*n* = 16) was the predominant resistance mechanism in 34.78% of the China TRAF isolates, followed by TR_34_/L98H (*n* = 11), TR_46_/Y121F/T289A (*n* = 3), G54R (*n* = 3), G54V (*n* = 2), TR_34_/L98H/S297T (*n* = 2), M220I (*n* = 2), G432A (*n* = 1), and nonsynonymous mutations (*n* = 6).

The geographic origin of the TRAF isolates appeared to concentrate in eastern and southeastern areas (Table 4). All isolates harboring TR_34_/L98H-related mutations exhibited high-level resistance to itraconazole (MIC, 8 to 16 mg/liter) and intermediate susceptibility or resistance to posaconazole and voriconazole, except for two TR_34_/L98H/S297T isolates, which had lower voriconazole MICs. In total, three voriconazole-resistant isolates harboring TR_46_/Y121F/T289A were identified so far, two from the environment and one from a patient.

As shown by microsatellite genotyping, STR typing of the Chinese TRAF isolates demonstrated two major clusters. Seven isolates with the TR_34_/L98H mutant type in China showed no genetic variability, suggesting a single and recent origin for these resistant isolates. Similarly, Abdolrasouli et al. (31) have described a similar structure in the TR-mediated azole-resistant A. fumigatus population in India. However, these observations contrast with the heterogeneity that was observed in environmental and clinical isolates in the Netherlands (32). The total of 13 Chinese isolates with TR_34_/L98H/S297T/F495I emerged from only one branch, notably an identical allelic profile with TR_34_/L98H/S297T/F495I, present in clinical and environmental A. fumigatus isolates from China, suggesting an environmental origin of this major resistance mechanism. The two groupings suggested that these isolates have different evolutionary sources than the major TR_34_/L98H complex. Our study confirmed that resistance due to TR_34_/L98H mutation among A. fumigatus isolates evolved from separate local isolates (33).

Our study was limited by the relatively small number of clinical A. fumigatus isolates included and the uneven geographic distribution in China. There are currently no azole resistance surveillance programs in China and many other countries, which would allow for more systematic collection and analysis of clinical A. fumigatus isolates. Furthermore, routine MIC testing is not performed in most clinical microbiology laboratories, which further complicates setting up such surveillance networks.

In conclusion, olorofim displays potent *in vitro* activity against A. fumigatus originating from China, including TRAF isolates. Further studies are needed to evaluate the *in vivo* efficacy of olorofim for the treatment of IA.

The need for novel targets is underscored by the increasing reports of TRAF both in patients and the environment. Despite multiple reports of TRAF in China, there is a need for systematic resistance surveillance to increase our understanding of resistance epidemiology and to guide antifungal treatment recommendations.

## MATERIALS AND METHODS

### *Aspergillus* isolates and species identification.

A total of 111 clinical A. fumigatus isolates were collected from Huashan Hospital, Fudan University, from 2012 to 2017 in Shanghai, China. The isolates were identified based on morphological features and sequence analysis of the partial *β-tubulin* gene (*benA*) sequences (7). The primers used are listed in Table S1 in the supplemental material. Isolate information and GenBank accession numbers for the generated *benA* sequences are listed in Table S2.

### Antifungal susceptibility testing.

*In vitro* antifungal susceptibility testing of the 111 isolates was performed according to the EUCAST definitive document (E.DEF 9.3.1). Olorofim was provided by F2G, Ltd. (Manchester, UK). Comparator antifungal agents, including amphotericin B, itraconazole, voriconazole, posaconazole, isavuconazole, anidulafungin, caspofungin, and micafungin, were purchased from Sigma-Aldrich (MO, USA). The testing ranges for olorofim, voriconazole, and micafungin were 0.008 to 8 mg/liter, 0.002 to 0.2 mg/liter, and 0.004 to 0.4 mg/liter, respectively. The ranges for amphotericin B, itraconazole, posaconazole, isavuconazole, anidulafungin, and caspofungin were 0.016 to 16 mg/liter. For olorofim, endpoints were determined after 48 h of incubation at 100% inhibition compared with the growth control.

Resistant isolates were defined according to the EUCAST breakpoints (version 10.0). There are no clinical breakpoints available for echinocandins and olorofim. Candida parapsilosis ATCC 22019 and C. krusei ATCC 6258 were used as the quality control strains.

### *cyp51A* gene sequencing.

Non-WT A. fumigatus isolates were selected for detection of *cyp51A* mutations. Genomic DNA was extracted as previously described (34), and full sequences of the *cyp51A* gene together with the promoter region were amplified and sequenced (35) (the primers used are listed in Table S1). The promoter and full sequence of *cyp51A* were aligned with the WT A. fumigatus strain (GenBank accession no. AF338659) using MAFFT version 7 (36). Tandem repeats (TR) in the gene promoter and mutations in the open reading frame were characterized after sequence alignment.

### Genotyping of A. fumigatus isolates.

Four azole-resistant A. fumigatus isolates were subjected to microsatellite typing, as previously described (37). Nine STR loci (STR *Af*2A, 2B, 2C, 3A, 3B, 3C, 4A, 4B, and 4C) were amplified in three separated multiplex PCRs. Each of the multiplex PCRs contained three different STRs. The fragments obtained were mixed with formamide and analyzed with GeneScan 500 LIZ on a 3730 DNA analyzer (Applied Biosystems, Foster City, CA, USA). The repeat numbers of the nine markers of all isolates were analyzed using Peak Scanner software 2 (Thermo Fisher, CA, USA).

### Genetic analysis of microsatellite genotypes.

To understand the genetic relationship of the azole-resistant A. fumigatus isolates in China to the global collection, a total of 29 Chinese azole-resistant A. fumigatus isolates (27 clinical and 2 environmental) and 102 azole-resistant A. fumigatus isolates collected globally were included by literature searching in PubMed. The twenty-nine Chinese azole-resistant A. fumigatus isolates included 25 isolates from the literature (7, 13, 15) and 4 isolates from the current study. The 102 azole-resistant A. fumigatus isolates were selected from the literature (6, 19, 38–46) as representative of different genotypes and geographic areas worldwide. The composite genotype for each of the 131 A. fumigatus isolates was identified based on alleles at all nine microsatellite loci. The genotype markers were then used to identify genetic relationships among isolates. Dendrograms were generated by the unweighted pair group method using average linkages implemented in BioNumerics 7.6 (bioMérieux). A minimum spanning tree was also calculated in BioNumerics 7.6 using advanced cluster analysis. Results of these analyses were used to infer the potential source(s) of the triazole-resistant clinical and environmental A. fumigatus isolates in China.

### Literature review.

A literature searching was carried out in databases including Pubmed/Medline, Scopus, Web of Science, Embase, and China National Knowledge Infrastructure (CNKI; https://www.cnki.net/). The English and Chinese language (CNKI database) literature between 1966 and 2020 was reviewed using search terms “China,” “Chinese,” “Aspergillus fumigatus,” “genetic diversity,” “short tandem repeats,” “STR,” “antifungal susceptibility,” “azole resistance,” and “fungicide resistance.”

### Data availability.

Accession numbers of 111 clinical strains in this study are listed in Table S2.

## Supplementary Material

Supplemental file 1
