# Supplementary material for: Potency of Olorofim (F901318) Compared to Contemporary Antifungal Agents against Clinical Aspergillus fumigatus Isolates and Review of Azole Resistance Phenotype and Genotype Epidemiology in China
Source: Antimicrob Agents Chemother. 2021 Apr 19;65(5):e02546-20. doi: 10.1128/AAC.02546-20 (PMC8092882; doi:10.1128/AAC.02546-20)
Supplement: Supplemental file 1 [file AAC.02546-20-s0001.pdf]

Table S1. Sequences of primers used in this study

Gene name	Primer name	Primer sequence	Function	PCR condition		
benA	370.1	AATTGGTGCCGCTTTCTGG	*benA* forward primer	95°C	7min	
	370.2	AGTTGTCGGGACGGAATAG	*benA* reverse primer		95℃	30s
				35 cycles	61℃	30s
					72℃	1 min
				72℃	10min	

Gene name	Primer name	Primer sequence	Function	PCR condition		
cyp51A	2.47	TCATATGTTGCTCAGCGG	*cyp51A* forward primer; *cyp51A* Sequencing primer	98℃	30s	
	5.2	TCGAGGGGTGAATTAAGTATAATACAC	*cyp51A* reverse primer; *cyp51A* Sequencing primer		98℃	10s
	2.48	ATGGTGCCGATGCTATGG	*cyp51A* Sequencing primer	40 cycles	58℃	30s
	2.49	CTGTCTCACTTGGATGTG	*cyp51A* Sequencing primer		72℃	4min
	2.50	TAGTCCATTGACGACCCC	*cyp51A* Sequencing primer	72℃	10min	
	2.52	TTGCTGGCCGTTTTTGTATGTGCAG	*cyp51A* Sequencing primer			
	2.58	TCTCTGCACGCAAAGAAGAAC	*cyp51A* Sequencing primer			
	5.24	GTGCTCCTTGCTTCACCTG	*cyp51A* Sequencing primer			

STable2. Isolates information and GenBank accession numbers for generated *benA* sequences of the 111 *A. fumigatus* isolates tested in this study

Strain No.	Species name	Accession No.	Source	Origin
246-60	*Aspergillus fumigatus*	MK190287	sputum	Shanghai China
246-62	*Aspergillus fumigatus*	MK190288	sputum	Shanghai China
246-63	*Aspergillus fumigatus*	MK190289	sputum	Shanghai China
246-64	*Aspergillus fumigatus*	MK190290	Catheter	Shanghai China
246-65	*Aspergillus fumigatus*	MK190291	sputum	Shanghai China
246-66	*Aspergillus fumigatus*	MK190292	sputum	Shanghai China
246-67	*Aspergillus fumigatus*	MK190293	sputum	Shanghai China
246-68	*Aspergillus fumigatus*	MK190294	sputum	Shanghai China
246-69	*Aspergillus fumigatus*	MK190295	substitution fluid	Shanghai China
246-70	*Aspergillus fumigatus*	MK190296	pleural fluid	Shanghai China
246-71	*Aspergillus fumigatus*	MK190297	puncture fluid	Shanghai China
246-72	*Aspergillus fumigatus*	MK190298	sputum	Shanghai China
246-73	*Aspergillus fumigatus*	MK190299	BALF	Shanghai China
246-74	*Aspergillus fumigatus*	MK190300	BALF	Shanghai China
246-76	*Aspergillus fumigatus*	MK190301	BALF	Shanghai China
246-77	*Aspergillus fumigatus*	MK190302	BALF	Shanghai China
246-78	*Aspergillus fumigatus*	MK190303	sputum	Shanghai China
246-81	*Aspergillus fumigatus*	MK190304	BALF	Shanghai China
247-01	*Aspergillus fumigatus*	MK190305	BALF	Shanghai China
247-02	*Aspergillus fumigatus*	MK190306	substitution fluid	Shanghai China
247-04	*Aspergillus fumigatus*	MK190307	sputum	Shanghai China
247-05	*Aspergillus fumigatus*	MK190308	BALF	Shanghai China
247-06	*Aspergillus fumigatus*	MK190309	sputum	Shanghai China
247-07	*Aspergillus fumigatus*	MK190310	sputum	Shanghai China
247-11	*Aspergillus fumigatus*	MK190311	BALF	Shanghai China
247-13	*Aspergillus fumigatus*	MK190312	sputum	Shanghai China
247-14	*Aspergillus fumigatus*	MK190313	sputum	Shanghai China
247-15	*Aspergillus fumigatus*	MK190314	sputum	Shanghai China
247-17	*Aspergillus fumigatus*	MK190315	sputum	Shanghai China
247-19	*Aspergillus fumigatus*	MK190316	articular cavity fluid	Shanghai China
247-20	*Aspergillus fumigatus*	MK190317	sputum	Shanghai China
247-21	*Aspergillus fumigatus*	MK190318	sputum	Shanghai China
247-22	*Aspergillus fumigatus*	MK190319	faeces	Shanghai China
247-25	*Aspergillus fumigatus*	MK190320	sputum	Shanghai China
247-26	*Aspergillus fumigatus*	MK190321	sputum	Shanghai China
247-27	*Aspergillus fumigatus*	MK190322	sputum	Shanghai China
247-29	*Aspergillus fumigatus*	MK190323	sputum	Shanghai China
247-31	*Aspergillus fumigatus*	MK190324	sputum	Shanghai China
247-32	*Aspergillus fumigatus*	MK190325	sputum	Shanghai China

Strain No.	Species name	Accession No.	Source	Origin
247-33	*Aspergillus fumigatus*	MK190326	sputum	Shanghai China
247-34	*Aspergillus fumigatus*	MK190327	sputum	Shanghai China
247-35	*Aspergillus fumigatus*	MK190328	sputum	Shanghai China
247-36	*Aspergillus fumigatus*	MK190329	sputum	Shanghai China
247-37	*Aspergillus fumigatus*	MK190330	sputum	Shanghai China
247-38	*Aspergillus fumigatus*	MK190331	sputum	Shanghai China
247-39	*Aspergillus fumigatus*	MK190332	sputum	Shanghai China
247-40	*Aspergillus fumigatus*	MK190333	sputum	Shanghai China
247-41	*Aspergillus fumigatus*	MK190334	sputum	Shanghai China
247-42	*Aspergillus fumigatus*	MK190335	sputum	Shanghai China
247-44	*Aspergillus fumigatus*	MK190336	sputum	Shanghai China
247-45	*Aspergillus fumigatus*	MK190337	sputum	Shanghai China
247-46	*Aspergillus fumigatus*	MK190338	urine	Shanghai China
247-47	*Aspergillus fumigatus*	MK190339	sputum	Shanghai China
247-48	*Aspergillus fumigatus*	MK190340	sputum	Shanghai China
247-49	*Aspergillus fumigatus*	MK190341	sputum	Shanghai China
247-50	*Aspergillus fumigatus*	MK190342	sputum	Shanghai China
247-51	*Aspergillus fumigatus*	MK190343	sputum	Shanghai China
247-52	*Aspergillus fumigatus*	MK190344	sputum	Shanghai China
247-53	*Aspergillus fumigatus*	MK190345	sputum	Shanghai China
247-54	*Aspergillus fumigatus*	MK190346	sputum	Shanghai China
247-55	*Aspergillus fumigatus*	MK190347	sputum	Shanghai China
247-56	*Aspergillus fumigatus*	MK190348	sputum	Shanghai China
247-57	*Aspergillus fumigatus*	MK190349	sputum	Shanghai China
247-61	*Aspergillus fumigatus*	MK190350	BALF	Shanghai China
247-62	*Aspergillus fumigatus*	MK190351	sputum	Shanghai China
247-63	*Aspergillus fumigatus*	MK190352	sputum	Shanghai China
247-64	*Aspergillus fumigatus*	MK190353	sputum	Shanghai China
247-65	*Aspergillus fumigatus*	MK190354	sputum	Shanghai China
247-66	*Aspergillus fumigatus*	MK190355	sputum	Shanghai China
247-67	*Aspergillus fumigatus*	MK190356	sputum	Shanghai China
247-68	*Aspergillus fumigatus*	MK190357	CSF	Shanghai China
247-69	*Aspergillus fumigatus*	MK190358	sputum	Shanghai China
247-70	*Aspergillus fumigatus*	MK190359	sputum	Shanghai China
247-71	*Aspergillus fumigatus*	MK190360	sputum	Shanghai China
247-72	*Aspergillus fumigatus*	MK190361	sputum	Shanghai China
247-73	*Aspergillus fumigatus*	MK190362	sputum	Shanghai China
247-75	*Aspergillus fumigatus*	MK190363	sputum	Shanghai China
247-76	*Aspergillus fumigatus*	MK190364	urine	Shanghai China
247-77	*Aspergillus fumigatus*	MK190365	sputum	Shanghai China
247-78	*Aspergillus fumigatus*	MK190366	sputum	Shanghai China
247-79	*Aspergillus fumigatus*	MK190367	sputum	Shanghai China

**Strain No.**	**Species name**	**Accession No.**	**Source**	**Origin**
247-80	*Aspergillus fumigatus*	MK190368	sputum	Shanghai China
248-01	*Aspergillus fumigatus*	MK190369	sputum	Shanghai China
248-02	*Aspergillus fumigatus*	MK190370	sputum	Shanghai China
248-03	*Aspergillus fumigatus*	MK190371	sputum	Shanghai China
248-04	*Aspergillus fumigatus*	MK190372	sputum	Shanghai China
248-05	*Aspergillus fumigatus*	MK190373	sputum	Shanghai China
248-06	*Aspergillus fumigatus*	MK190374	CSF	Shanghai China
248-07	*Aspergillus fumigatus*	MK190375	sputum	Shanghai China
248-08	*Aspergillus fumigatus*	MK190376	sputum	Shanghai China
248-09	*Aspergillus fumigatus*	MK190377	sputum	Shanghai China
248-10	*Aspergillus fumigatus*	MK190378	sputum	Shanghai China
248-11	*Aspergillus fumigatus*	MK190379	sputum	Shanghai China
248-13	*Aspergillus fumigatus*	MK190380	Catheter	Shanghai China
248-14	*Aspergillus fumigatus*	MK190381	pleural fluid	Shanghai China
248-16	*Aspergillus fumigatus*	MK190382	BALF	Shanghai China
248-18	*Aspergillus fumigatus*	MK190383	sputum	Shanghai China
248-19	*Aspergillus fumigatus*	MK190384	sputum	Shanghai China
248-20	*Aspergillus fumigatus*	MK190385	sputum	Shanghai China
248-21	*Aspergillus fumigatus*	MK190386	sputum	Shanghai China
248-22	*Aspergillus fumigatus*	MK190387	sputum	Shanghai China
248-23	*Aspergillus fumigatus*	MK190388	BALF	Shanghai China
248-24	*Aspergillus fumigatus*	MK190389	sputum	Shanghai China
248-25	*Aspergillus fumigatus*	MK190390	sputum	Shanghai China
248-26	*Aspergillus fumigatus*	MK190391	sputum	Shanghai China
248-28	*Aspergillus fumigatus*	MK190392	sputum	Shanghai China
248-29	*Aspergillus fumigatus*	MK190393	sputum	Shanghai China
248-30	*Aspergillus fumigatus*	MK190394	BALF	Shanghai China
248-32	*Aspergillus fumigatus*	MK190395	sputum	Shanghai China
248-33	*Aspergillus fumigatus*	MK190396	sputum	Shanghai China
248-34	*Aspergillus fumigatus*	MK190397	sputum	Shanghai China

BAL: bronchial alveolar lavage fluid

CSF: cerebrospinal fluid
